# Reduced versus standard dose apixaban for secondary prevention of cancer-associated venous thromboembolism: A systematic review and meta-analysis

**DOI:** 10.3389/fonc.2025.1690984

**Published:** 2025-12-19

**Authors:** Vasu Malhotra, Shreya G. Patel, Ardit Feinaj, Devesh Amin, Henry Ash, Mohammad B. Boozo, Zachary Breslow, Jennifer Trube, Muhammad Z. Farooq, Michael Sabina

**Affiliations:** 1 Department of Internal Medicine, Lakeland Regional Health, Lakeland, FL, United States; 2 Department of Internal Medicine, Southern Illinois University School of Medicine, Springfield, IL, United States; 3 Department of Hematology & Oncology, Lakeland Regional Health, Lakeland, FL, United States

**Keywords:** apixaban, VTE (venous thromboembolism), cancer-associated thrombosis, anticoagulation, meta-analysis, bleeding risk and anticoagulation, direct oral anticoagulants (DOA), extended anticoagulation

## Abstract

Patients with cancer-associated venous thromboembolism (VTE) often require prolonged anticoagulation because malignancy and chemotherapy can persist for months to years, sustaining both thrombotic and bleeding risks. While full-dose apixaban (5 mg twice daily) is recommended for secondary prevention, extended use may increase bleeding in this vulnerable population. Reducing the dose to 2.5 mg twice daily after six months of full-dose treatment has been proposed to lower bleeding risk without compromising protection against recurrent VTE. To clarify the safety and efficacy of this strategy, we conducted a meta-analysis of randomized controlled trials comparing reduced- versus standard-dose apixaban for extended therapy in patients with cancer-associated VTE. The study followed PRISMA guidelines and was registered in PROSPERO (CRD420251026337). Hazard ratios and risk ratios with 95% confidence intervals were pooled using a random-effects model, and evidence certainty was assessed with GRADE. Two randomized trials comprising 2,126 patients (EVE, n = 360; API-CAT, n = 1,766) met inclusion criteria. The composite outcome of recurrent VTE with bleeding showed a significantly lower risk with reduced-dose apixaban (risk ratio 0.79, 95% CI 0.65–0.96; I^2^ = 0%), and the composite of major and clinically relevant nonmajor bleeding was also reduced (HR 0.75 (95% CI 0.63–0.88; I^2^ = 0%). No significant differences were observed for recurrent VTE, major bleeding, clinically relevant nonmajor bleeding, mortality, deep-vein thrombosis, or pulmonary embolism. These findings indicate that reduced-dose apixaban after the initial six months of anticoagulation decreases bleeding without loss of efficacy, supporting dose de-escalation as a safe, evidence-based approach for extended anticoagulation in cancer-associated VTE.

**Systematic review registration:**

https://www.crd.york.ac.uk/prospero/, identifier PROSPERO CRD420251026337.

## Introduction

Hypercoagulability is a well-recognized hallmark of malignancy and remains one of the most clinically significant complications among patients with active cancer. The interplay of tumor-derived procoagulant activity, cytokine-mediated inflammation, and endothelial dysfunction contributes to a prothrombotic environment that predisposes to both venous and arterial thromboses, including deep vein thrombosis (DVT), pulmonary embolism (PE), myocardial infarction, and stroke (1). In addition to intrinsic tumor biology, treatment-related factors such as chemotherapy, hormonal therapy, surgery, and indwelling venous catheters further amplify thrombotic risk (2). This prothrombotic tendency not only increases the likelihood of a first venous thromboembolic event (VTE) but also confers a substantial risk of recurrence. Following an initial episode of cancer-associated VTE, the six-month recurrence rate has been estimated at approximately 5.1%, and recurrent PE in particular is associated with the highest short-term mortality (3). These recurrent events significantly worsen prognosis, with affected patients demonstrating nearly a twofold increase in all-cause mortality compared with those without recurrence (3).

To mitigate these risks, the American Society of Clinical Oncology (ASCO) and the American Society of Hematology (ASH) recommend extended anticoagulation beyond the conventional 3 to 6-month treatment window for patients with active malignancy or those receiving ongoing cancer therapy (4, 5). These recommendations are primarily based on expert consensus and limited evidence from prospective studies such as DALTECAN and TiCAT, which evaluated the feasibility of prolonged low-molecular-weight heparin (LMWH) therapy in selected oncology populations (6–10). Despite these foundational efforts, the long-term use of LMWH remains burdensome and costly, leading to a shift toward direct oral anticoagulants (DOACs) as preferred alternatives. Apixaban, a selective factor Xa inhibitor, has emerged as one of the most widely studied and utilized DOACs in this population. Multiple randomized controlled trials (RCTs), including CARAVAGGIO, SELECT-D, and ADAM VTE, demonstrated that apixaban and related Xa inhibitors are at least as effective as LMWH in preventing recurrent VTE while maintaining comparable or improved bleeding profiles (7, 11, 12). As a result, both ASCO and the International Society on Thrombosis and Haemostasis (ISTH) have endorsed apixaban as a first-line option for the treatment of cancer-associated thrombosis (6, 9).

The clinical uncertainty surrounding the optimal apixaban dose beyond six months remains a critical evidence gap with direct implications for patient safety and quality of life. In practice, many clinicians empirically reduce anticoagulation intensity after the initial treatment period, despite the paucity of high-quality data to guide this approach. A rigorous synthesis of the available randomized evidence is therefore warranted to clarify the relative efficacy and safety of reduced-dose versus standard-dose apixaban in this setting. The present pooled analysis integrates data from the Extended Venous Thromboembolism Treatment with Apixaban in Cancer (EVE) and Apixaban in Cancer Associated Thrombosis (API-CAT) trials, the only two randomized controlled trials to directly compare these dosing regimens in patients with cancer-associated VTE (13, 14). We hypothesized that reduced-dose apixaban (2.5 mg twice daily) would maintain comparable protection against recurrent thromboembolism while reducing the incidence of major and clinically relevant non-major bleeding during the extended phase of anticoagulation.

## Methods

### Study design

We performed this meta-analysis according to the Preferred Reporting Items for Systematic Reviews and Meta-Analyses (PRISMA) guidelines (15). We registered the protocol for this study on the International Prospective Register of Systematic Reviews (PROSPERO) (registration ID: CRD420251026337).

### Search strategy

The literature search was conducted across five databases: PubMed-MEDLINE, Embase-OVID, the Cochrane Central Register of Controlled Trials (CENTRAL), ClinicalTrials.gov, and Web of Science. All databases were searched from inception through November 5, 2025, without language restrictions. Search terms combined Medical Subject Headings (MeSH) and free-text keywords related to venous thromboembolism, cancer, anticoagulation, and apixaban. Detailed line-by-line search strategies for each database, along with the date last run, are provided in the **Supplementary Materials**. Reference lists of included articles and prior systematic reviews were manually screened to identify any additional eligible studies. Grey literature sources, including conference abstracts and dissertations, were not searched, as our objective was restricted to peer-reviewed randomized controlled trials with complete outcome data.

### Eligibility criteria

Data extraction was performed manually by two investigators using a standardized template, with discrepancies resolved by a third investigator as needed. The two authors defined eligibility criteria according to the PICOS framework. Studies were eligible if they were randomized controlled trials (RCTs) enrolling adult patients with cancer-associated VTE requiring extended anticoagulation, and if they directly compared RD (2.5 mg twice daily) versus FD (5 mg twice daily) apixaban. Additionally, studies were eligible if they reported recurrent VTE and bleeding as outcomes and had a minimum follow-up period of at least six months to ensure consistency across studies. Conversely, studies were excluded if they focused on initial treatment dosing (i.e., the first six months after VTE diagnosis), assessed anticoagulants other than apixaban, lacked adjudicated clinical outcomes, or did not provide a direct dose comparison.

### Study selection and data extraction

Data extraction and study screening were performed independently by two investigators using Rayyan to facilitate blinded review and conflict resolution (16). A standardized data extraction template was employed, and any discrepancies were resolved by consensus with a third investigator. The following information was extracted: study identifier (author[s], publication year, or trial name), study design, dosing strategy, follow-up duration, population sample, and primary and secondary outcomes. Clinical outcomes included the composite of recurrent VTE and major bleeding, the composite of major and clinically relevant non-major bleeding (CRNMB), and each outcome individually (DVT, PE, and all-cause mortality). When applicable, outcomes were defined according to the International Society on Thrombosis and Haemostasis (ISTH) criteria. Baseline characteristics were also collected, including patient age, sex, body mass index, history of VTE, cancer type (e.g., breast, prostate, colorectal, lung, and other), and VTE presentation (PE with or without DVT versus isolated DVT).

A completed PRISMA 2020 checklist and the study-level dataset used for meta-analytic calculations are available in the **Supplementary Materials**. All meta-analyses and forest plots were generated using RevMan version 5.5, and the CSV file containing the extracted data entered into RevMan is provided to ensure full reproducibility (16).

### Risk of bias

The risk of bias for each included study was assessed using the Revised Cochrane Risk of Bias Tool (RoB 2.0), which examines five domains: the randomization process, deviations from intended interventions, missing outcome data, outcome measurement, and selection of reported results. Studies were subsequently classified as having a low, moderate, or high overall risk of bias. The certainty of evidence for each outcome was evaluated using the Grading of Recommendations, Assessment, Development, and Evaluation (GRADE) framework, which considers risk of bias, inconsistency, indirectness, imprecision, and publication bias. Each outcome was then rated as high, moderate, low, or very low certainty. Publication bias was not assessed using Egger’s test, in accordance with Cochrane Handbook guidance, as fewer than 10 studies were included, below the threshold required for reliable interpretation of such tests (17). Full details of the risk of bias assessment and GRADE evidence profiles are provided in the Supplementary File. Risk-of-bias assessments and certainty-of-evidence ratings were conducted in accordance with the Cochrane Handbook for Systematic Reviews of Interventions (18).

### Statistical analysis

All meta-analyses were performed using RevMan version 5.5 (16). Statistical analyses employed a random-effects model to estimate pooled hazard ratios (HRs) and risk ratios (RRs) with corresponding 95% confidence intervals (CIs). The Mantel–Haenszel method was used for pooling RRs, while between-study variance was estimated using the DerSimonian–Laird method. The Knapp–Hartung adjustment was applied for HRs to improve variance precision. Heterogeneity was assessed using Cochran’s Q test, quantified by the I² statistic (with I² >50% indicating substantial heterogeneity), and further characterized by the between-study variance parameter (τ²).

## Results

### Search results

**Figure 1:**
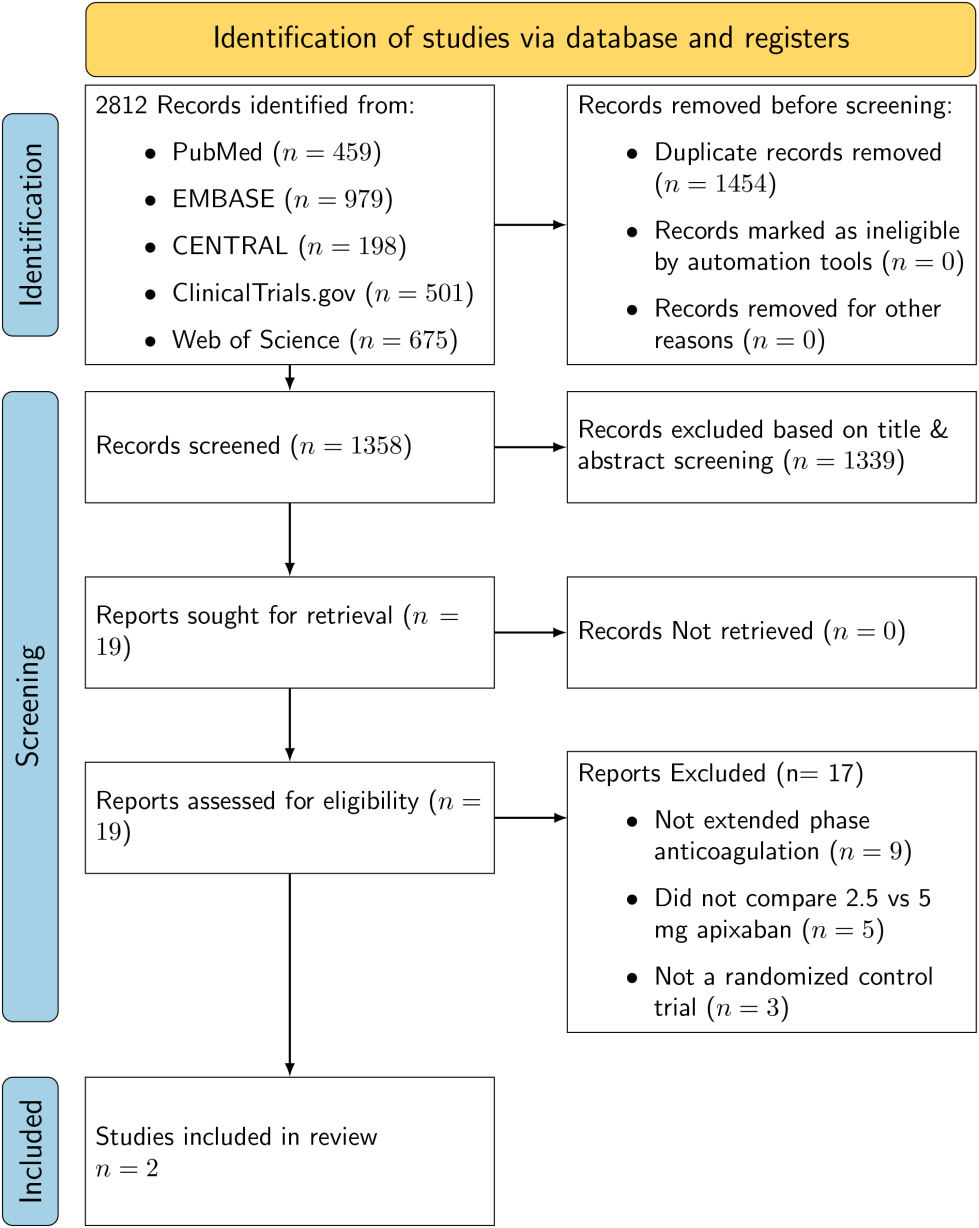
PRISMA flow diagram outlining the study identification, screening, and selection process for inclusion in the meta-analysis.

A total of 2,812 records were identified across all databases and trial registries, including 459 from PubMed/MEDLINE, 979 from Embase, 198 from CENTRAL, 501 from ClinicalTrials.gov, and 675 from Web of Science. After removing 1,454 duplicate records, 1,358 unique records remained for title and abstract screening. Of these, 1,339 records were excluded as irrelevant to the study objective, leaving 19 full-text articles for detailed assessment. Following eligibility review, two randomized controlled trials met the inclusion criteria for this pooled analysis: Extended Venous Thromboembolism Treatment with Apixaban in Cancer (EVE) by McBane et al. (2024) and Apixaban in Cancer Associated Thrombosis (API-CAT) by Mahé et al. (2025). Both trials compared reduced-dose apixaban (2.5 mg twice daily) with standard-dose apixaban (5 mg twice daily) for extended-phase anticoagulation in patients with cancer-associated venous thromboembolism. The PRISMA 2020 flow diagram summarizing the study selection process is presented in **Figure 1** (13, 14).

### Characteristics of included studies

Both the EVE 2024 and the API-CAT 2025 were randomized, double-blind, controlled studies that directly compared reduced-dose apixaban (2.5 mg twice daily) with standard-dose apixaban (5 mg twice daily) for the extended treatment of cancer-associated venous thromboembolism (VTE). The EVE trial, conducted by McBane et al. (2024), enrolled 360 patients with active cancer who had completed at least six months of anticoagulation for VTE and were randomized to receive either reduced-dose or standard-dose apixaban for an additional 12 months. The API-CAT trial, reported by Mahé et al. (2025), was a larger, phase III study that included 1,766 patients under similar inclusion criteria and compared the same dosing regimens over a 12-month extended-treatment period. Together, the two trials included 2,126 patients, with 358 participants in the EVE trial and 1,768 in the API-CAT trial. In total, 1,045 patients received the reduced dose, and 1,081 received the standard dose. Both studies used blinded adjudication of efficacy and safety outcomes, including recurrent VTE and major or clinically relevant non-major bleeding. Median follow-up duration was 12 months in both trials. While the overall designs were closely aligned, the API-CAT trial provided greater statistical precision because of its larger sample size and broader international representation, whereas the EVE trial contributed complementary data from a U.S. multicenter cohort. The methodological consistency between the two studies supported the validity of pooling their data for quantitative analysis. Key characteristics of the included trials, including study design, sample size, dosing comparisons, follow-up duration, patient populations, and definitions of primary, secondary, and composite outcomes, are summarized in **Table 1** (13, 14).

**Table 1:** Characteristics of included studies.

Study ID	Design	Sample size	Dosing compared	Follow-up	Population	Primary outcome	Secondary outcomes
EVE 2024	RCT, double-blind, multicenter	360 (179 reduced, 181 standard)	2.5 mg vs 5 mg twice daily after 6 months of anticoagulation	12 months	Patients with active cancer who completed ≥6 months of anticoagulation for VTE	Composite of major bleeding and clinically relevant non-major bleeding (CRNMB)	Recurrent VTE, arterial thromboembolism, composite of recurrent VTE plus bleeding
API-CAT 2025	Phase III RCT, double-blind, multicenter	1,766 (866 reduced, 900 standard)	2.5 mg vs 5 mg twice daily after ≥6 months of anticoagulation	12 months	Patients with active cancer and proximal deep-vein thrombosis or pulmonary embolism receiving extended therapy	Composite of recurrent VTE and major or clinically relevant non-major bleeding (CRNMB)	Individual components of the primary composite (recurrent VTE and bleeding events), all-cause death, arterial thromboembolism

CRNMB, clinically relevant non-major bleeding; DVT, deep-vein thrombosis; PE, pulmonary embolism; RCT, randomized controlled trial; VTE, venous thromboembolism; API-CAT, Apixaban in Cancer Associated Thrombosis; EVE, Extended Venous Thromboembolism Treatment with Apixaban in Cancer; HR, hazard ratio; RR, risk ratio; CI, confidence interval.

### Baseline characteristics

Across the two randomized controlled trials, a total of 2,126 patients with cancer-associated venous thromboembolism (VTE) were enrolled. The mean age ranged from 64 years in the EVE trial to 67 years in the API-CAT trial, with 43–47% male participants. Nearly all participants had active cancer at enrollment, and metastatic disease was present in approximately 60–66%. Performance status was favorable overall, with over 90% of patients reporting an ECOG 0–1. The API-CAT trial enrolled a broader international population, while EVE was primarily U.S.-based. The index event leading to anticoagulation was pulmonary embolism in roughly half of EVE participants and three-quarters of API-CAT participants, reflecting minor differences in case mix. The predominant cancer types were breast, gastrointestinal, lung, genitourinary, and gynecologic, consistent with the distribution typically observed in cancer-associated thrombosis. The median time from the index VTE to randomization was eight months in both trials. Together, these populations represent a contemporary cohort of ambulatory cancer patients with sustained thrombosis risk despite initial anticoagulation, supporting the pooled analysis of reduced- versus standard-dose apixaban in extended therapy. Baseline characteristics of participants in the EVE and API-CAT trials are summarized in **Table 2**.

**Table 2:** Baseline characteristics of included studies.

Characteristic	EVE Trial (n = 360)	API-CAT Trial (n = 1,766)
Mean age, years	64 ± 11	67 ± 11
Male sex, %	47	43.4
ECOG performance status 0–1, %	95	92.6
Active cancer, %	100	99.7
Metastatic disease, %	59.7	65.6
Concurrent systemic therapy, %	74	55
Creatinine clearance < 50 mL/min, %	Not reported	13.7
Qualifying index event	53% PE; 40% DVT; 6% atypical (VTE in other venous beds)	75.5% PE; 24.5% DVT
Most common tumor sites	GI (26%), hematologic (14%), lung (12%), gynecologic (10%)	Breast (23%), colorectal (15%), gynecologic (12%), lung (11%), prostate (9%)
Median time since index VTE, months	8 (IQR 6–12)	8 (IQR 6.5–12.6)
Follow-up duration, months	12	12

DVT, deep-vein thrombosis; ECOG, Eastern Cooperative Oncology Group performance status; GI, gastrointestinal; IQR, interquartile range; PE, pulmonary embolism; VTE, venous thromboembolism; API-CAT, Apixaban in Cancer Associated Thrombosis; EVE, Extended Venous Thromboembolism Treatment with Apixaban in Cancer.

### Outcomes

**Figure 2:**
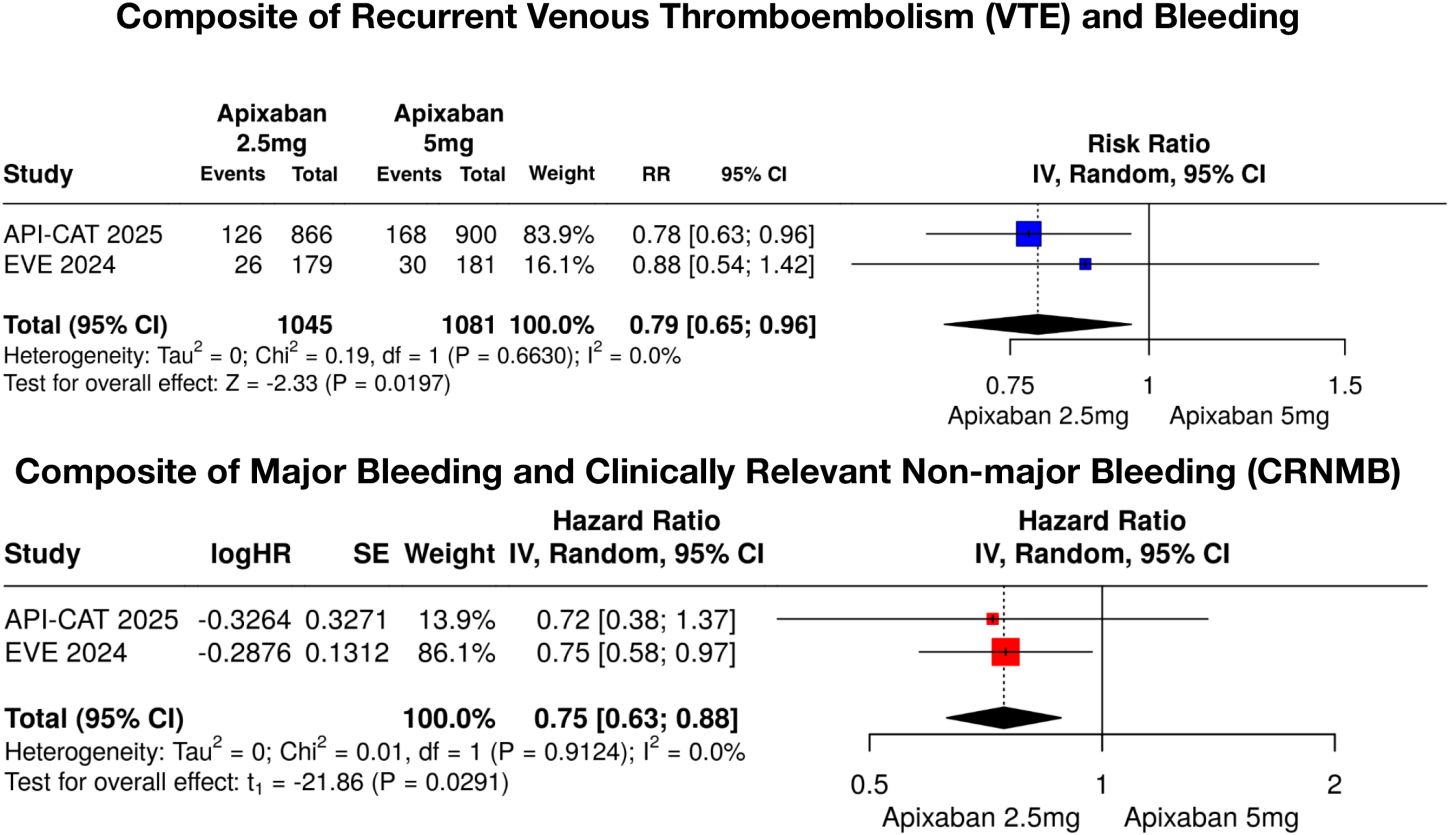
Pooled hazard ratios for composite outcomes. Top: Composite of recurrent venous thromboembolism (VTE) and bleeding. Bottom: Composite of major bleeding and clinically relevant non-major bleeding (CRNMB). Pooled using random-effects model with inverse variance weighting. RR < 1.0 favors reduced-dose apixaban (2.5 mg twice daily). VTE, venous thromboembolism; CRNMB, clinically relevant non-major bleeding; CI, confidence interval; HR, hazard ratio.

**Figure 3:**
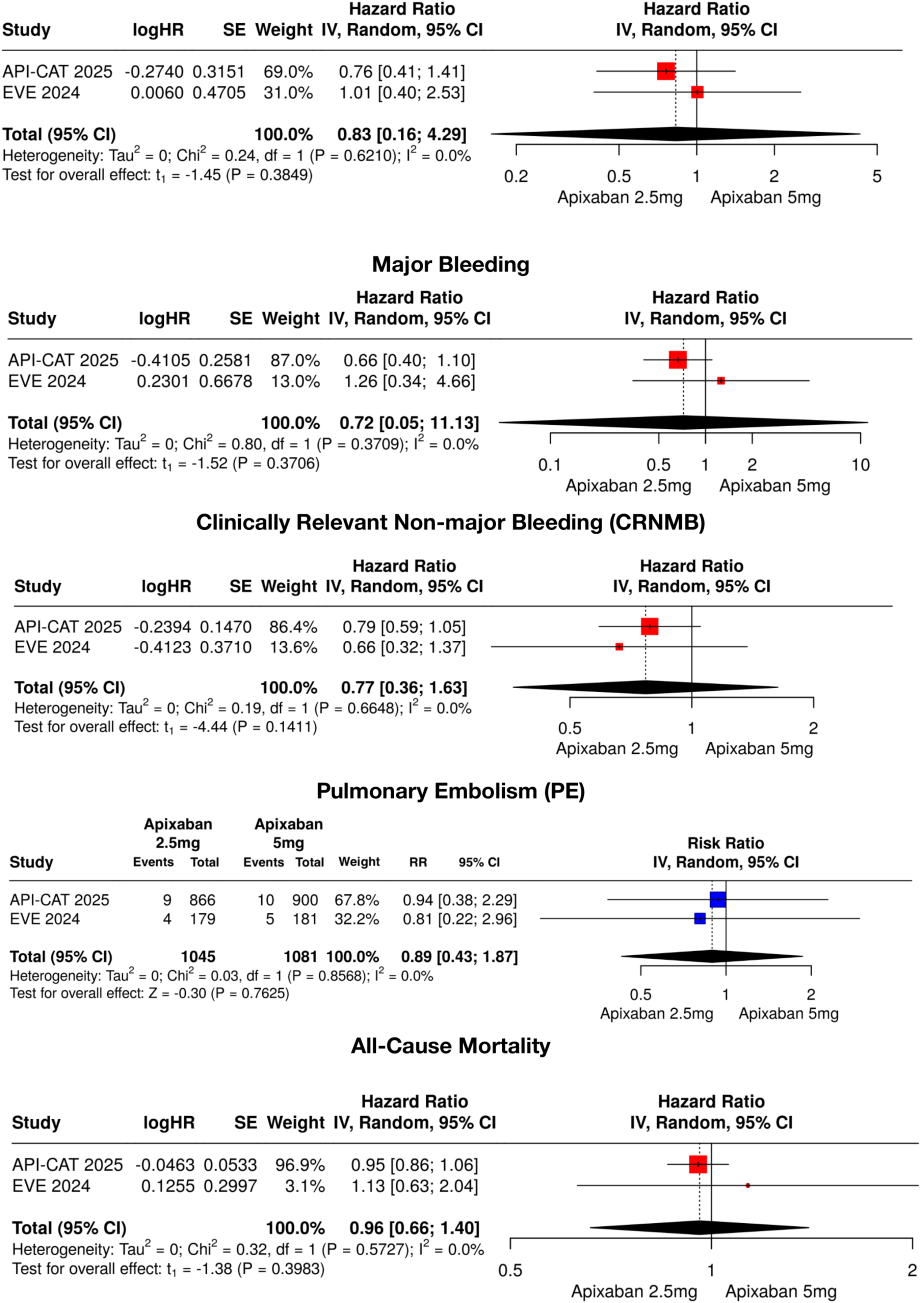
Pooled hazard ratios and risk ratios for individual outcomes. Individual analyses include recurrent VTE, major bleeding, clinically relevant non-major bleeding (CRNMB), pulmonary embolism (PE), deep-vein thrombosis (DVT), and all-cause mortality. Pooled estimates were calculated using random-effects models. RR < 1.0 favors reduced-dose apixaban (2.5 mg twice daily). VTE, venous thromboembolism; CRNMB, clinically relevant non-major bleeding; PE, pulmonary embolism; DVT, deep vein thrombosis; CI, confidence interval; HR, hazard ratio.

For the composite outcomes, the pooled RR for recurrent venous thromboembolism (VTE) with bleeding was 0.79 (95% CI, 0.65–0.96; I² = 0%), indicating a significant reduction in combined thrombotic and bleeding events. The composite of major and clinically relevant nonmajor bleeding showed a pooled HR 0.75 (95% CI 0.63–0.88; I² = 0%), reflecting a consistent decrease in overall bleeding risk across studies. The pooled results for the composite efficacy and safety outcomes are shown in **Figure 2**, and the pooled results for individual outcomes are presented in **Figure 3**.

Among individual outcomes, the pooled HRs for recurrent VTE (0.83, 95% CI, 0.16–4.29; I² = 0%), major bleeding (0.72, 95% CI, 0.05–11.13; I² = 0%), clinically relevant nonmajor bleeding (0.77, 95% CI, 0.36–1.63; I² = 0%), and all-cause mortality (0.96, 95% CI, 0.66–1.40; I² = 0%) demonstrated no statistically significant differences between groups. For VTE components, the pooled RRs for deep-vein thrombosis (0.95, 95% CI, 0.44–2.08; I² = 0%) and pulmonary embolism (0.89, 95% CI, 0.43–1.87; I² = 0%) similarly showed no significant differences. A detailed summary of pooled effect estimates and individual study-level data is presented in **Table 3**.

**Table 3:** Summary of pooled outcomes.

	EVE 2024			API-CAT 2025			Overall		
Endpoint	Apixaban 2.5mg (n=179)	Apixaban 5mg (n=181)	HR or RR (95% CI)	Apixaban 2.5mg (n=866)	Apixaban 5mg (n=900)	HR or RR (95% CI)	Apixaban 2.5mg (n=1045)	Apixaban 5mg (n=1081)	HR or RR (95% CI)
Composite Outcomes									
*Recurrent VTE and Bleed*	26 (14.5)	30 (16.6)	0.88 (0.54-1.42)	126 (14.5)	168 (18.7)	0.78 (0.75 - 0.96)	152 (14.5)	198 (18.3)	0.79 (0.65 - 0.96)
*Major bleeding episode and Clinically relevant nonmajor bleeding*	16 (8.9)	22 (12.2)	0.72 (0.38-1.37)	102 (12.1)	136 (15.6)	0.75 (0.58–0.97)	118 (11.3)	158 (14.6)	0.75 (0.63–0.88)
Individual outcomes									
*Recurrent Venous thromboembolism — no. (%)*	9 (5.0)	8 (4.4)	1.00 (0.40-2.53)	18 (2.1)	24 (2.8)	0.76 (0.41–1.41)	27 (2.6)	32 (3.0)	0.83 (0.16 - 4.29)
*Deep-vein thrombosis — no. (%)*	3 (1.7)	4 (2.2)	0.76 (0.17-3.34)	9 (1.1)	9 (1.1)	1.04 (0.41-2.61)	12 (1.1)	13 (1.2)	0.95 (0.44-2.08)
*Pulmonary embolism — no. (%)†*	4 (2.2)	5 (2.8)	0.81 (0.22-2.96)	9 (1.1)	10 (1.2)	0.94 (0.38-2.29)	13 (1.2)	15 (1.4)	0.89 (0.43-1.87)
*Major bleeding episode — no. (%)*	5 (2.8)	4 (2.2)	1.26 (0.34-4.66)	24 (2.9)	37 (4.3)	0.66 (0.40–1.10)	29 (2.8)	41 (3.8)	0.72 (0.05 - 11.13)
*Clinically relevant nonmajor bleeding — no. (%)¶*	12 (6.7)	18 (9.9)	0.66 (0.32-1.37)	84 (10.0)	107 (12.3)	0.79 (0.59–1.05)	96 (9.2)	125 (11.6)	0.77 (0.36 - 1.63)
*Death from any cause — no. (%)*	24 (13)	21 (12)	1.14 (0.75-2.04)	148 (17.7)	168 (19.6)	0.96 (0.86–1.06)	172 (16.5)	189 (17.5)	0.96 (0.66 - 1.40)

CI, confidence interval.

CRNMB, clinically relevant non-major bleeding; DVT, deep-vein thrombosis; HR, hazard ratio; PE, pulmonary embolism; RR, risk ratio; VTE, venous thromboembolism. Composite outcomes include recurrent VTE with bleeding and the combined endpoint of major bleeding plus CRNMB. Individual outcomes include recurrent VTE, DVT, PE, major bleeding, CRNMB, and all-cause mortality. Hazard or risk ratios less than 1.0 favor reduced-dose apixaban (2.5 mg twice daily) compared with standard-dose apixaban (5 mg twice daily). Pooled estimates were calculated using a random-effects model.

### Heterogeneity

Between-study heterogeneity was assessed using the I² and τ² statistics, both of which were 0% across all outcomes, indicating complete consistency between the included trials. Despite the absence of heterogeneity, several outcomes demonstrated wide confidence intervals due to the application of the Knapp–Hartung adjustment, which provides more conservative variance estimates in random-effects meta-analyses. Given that only two studies were included, the Knapp–Hartung method substantially widened the confidence intervals to account for uncertainty in between-study variance, reflecting limited precision rather than true variability between trials.

### Risk of bias assessment and certainty of evidence

Both API-CAT 2025 and EVE 2024 demonstrated low to moderate overall risk of bias. Randomization, allocation concealment, and blinding were appropriately performed in both studies, minimizing performance and detection bias. Outcome assessment was objective and centrally adjudicated, ensuring reliability. Attrition was minimal, and intention-to-treat analyses were used. The only minor concern was in the EVE trial, where limited sample size and broad interpretation of secondary outcomes could introduce selective reporting bias. Full RoB 2.0 domain evaluations and justifications are available in the Supplementary File (**Supplementary Table S1**). Certainty of evidence for each outcome was evaluated using the GRADE framework, considering risk of bias, inconsistency, indirectness, imprecision, and publication bias. Overall, most outcomes were rated as high to moderate certainty, with downgrades primarily due to imprecision from wide confidence intervals and limited study number. A detailed breakdown of the GRADE assessment for each outcome is provided in **Supplementary Table S2** (Supplementary File).

## Discussion

This pooled analysis evaluated reduced-dose versus standard-dose apixaban for extended anticoagulation in patients with cancer-associated venous thromboembolism (VTE), integrating data from the two available randomized controlled trials: the EVE and API-CAT studies (13, 14). Both trials demonstrated that reducing the apixaban dose to 2.5 mg twice daily after an initial six months of treatment preserved efficacy for preventing recurrent thromboembolism while significantly decreasing bleeding risk. The API-CAT trial established comparable efficacy of the reduced dose for recurrent VTE and superiority for major and clinically relevant non-major bleeding (CRNMB) (14). Similarly, the EVE trial observed a consistent trend toward fewer bleeding events without an increase in thrombotic recurrence (13). Collectively, these results indicate that a reduced-dose regimen offers an optimal balance between efficacy and safety in the extended treatment phase of cancer-associated thrombosis.

Our pooled findings indicate that reduced-dose apixaban provides comparable protection against recurrent thromboembolism relative to the standard dose. Rates of recurrent VTE, deep-vein thrombosis (DVT), pulmonary embolism (PE), and all-cause mortality were statistically similar between treatment arms, suggesting no meaningful difference in efficacy. In contrast, the composite of major bleeding and CRNMB occurred significantly less often with the reduced dose. Although CRNMB events are typically nonfatal, they are clinically relevant because they often lead to temporary discontinuation of therapy, additional medical evaluation, and increased patient anxiety. In oncology populations, where cumulative treatment toxicity and bleeding risk are already substantial, reducing such events without compromising thrombotic protection represents an important clinical advantage.

When interpreted in the broader context of direct oral anticoagulant (DOAC) therapy for cancer-associated VTE, these findings align with evidence from earlier trials evaluating rivaroxaban and edoxaban. The SELECT-D trial demonstrated that rivaroxaban was non-inferior to low-molecular-weight heparin (LMWH) for preventing recurrent VTE but was associated with a higher risk of gastrointestinal bleeding (7). Similarly, the Hokusai VTE Cancer trial found that edoxaban provided comparable protection against recurrence but carried an elevated bleeding risk in patients with mucosal malignancies (8). These observations contrast with apixaban’s more favorable bleeding profile, as demonstrated in CARAVAGGIO and ADAM VTE, which established apixaban as a preferred DOAC for cancer-associated thrombosis (11, 12).The consistency of the EVE and API-CAT findings with these earlier trials strengthens the evidence supporting apixaban’s use in extended therapy and distinguishes it as the only DOAC with randomized data supporting dose de-escalation after the initial treatment phase. This distinction underscores a unique therapeutic advantage for tailoring anticoagulation intensity in oncology patients who remain vulnerable to both thrombotic and hemorrhagic complications during long-term management.

The findings from this analysis support the concept of dose de-escalation after the initial 6-month treatment period, particularly in patients who remain at risk for thrombosis but have an elevated bleeding risk or are receiving concurrent cytotoxic therapy. Extending anticoagulation with a lower apixaban dose aligns with the broader movement toward individualized anticoagulation strategies in cancer care. In clinical practice, this approach may improve patient adherence and quality of life while reducing emergency visits, transfusions, and healthcare utilization related to bleeding. Furthermore, by minimizing interruptions in anticoagulation, a reduced-dose strategy could indirectly lower the risk of recurrent events associated with premature treatment cessation.

Despite the consistency between the two trials, some caution is warranted in generalizing these results to all cancer populations. Both EVE and API-CAT primarily enrolled ambulatory patients with solid tumors and preserved performance status, limiting applicability to those with hematologic malignancies, advanced frailty, or severe renal impairment. Future research should explore whether similar safety and efficacy outcomes extend to these higher-risk subgroups and whether dose-reduction strategies remain effective beyond 12 months of therapy. In summary, this pooled analysis demonstrates that reduced-dose apixaban provides comparable efficacy to the standard dose while significantly lowering the risk of clinically relevant bleeding. These results support dose reduction as a rational and evidence-based option for the extended-phase management of cancer-associated VTE, offering clinicians a safe and effective strategy to sustain anticoagulation in this complex patient population.

### Limitations

This analysis should be interpreted within the context of several methodological and clinical constraints. The size and scope of the API-CAT trial play a major role in shaping the limitations of this pooled analysis. Given its considerably larger sample size compared with the EVE trial, API-CAT contributed the majority of participants and, therefore, exerted greater statistical weight on the pooled estimates (14). This imbalance inevitably means that the overall summary effects reflect the characteristics and outcomes of API-CAT more strongly than those of EVE. Although both trials were randomized, double-blind, and methodologically aligned in dosing strategy, inclusion criteria, and follow-up duration, subtle differences in geographic representation, cancer distribution, and concomitant therapies could still influence the combined effect sizes (13, 14). The dominance of a single trial also reduces the ability to explore true between-study heterogeneity or perform meaningful subgroup analyses. This structural limitation underscores the need for additional randomized studies to validate these findings across diverse cancer populations and healthcare settings, which would provide a more balanced evidence base for future meta-analyses.

Beyond the disproportionate influence of API-CAT, the limited number of available randomized controlled trials constrains the breadth of this evidence synthesis. With only two eligible RCTs, the pooled analysis cannot fully account for variations across tumor types, disease stages, or ongoing treatment regimens, all of which can alter both thrombosis and bleeding risk profiles (4, 5). While both studies enrolled patients who had completed at least six months of anticoagulation without major complications, this criterion inherently selected a more stable and lower-risk population. As a result, the generalizability of these findings to patients with newly diagnosed malignancy, advanced disease burden, or early treatment-related toxicity remains uncertain. Expanding future investigations to include a broader spectrum of clinical severity and comorbidity would help contextualize these results within real-world oncology practice (3, 6).

The duration of follow-up represents another key limitation. Both trials evaluated outcomes over a 12-month period after randomization, offering valuable insight into the intermediate-term safety and efficacy of reduced-dose apixaban but leaving longer-term effects unaddressed (13, 14). Cancer-associated thrombosis is often a chronic condition that parallels the course of malignancy, and the risks of both recurrent VTE and bleeding may evolve over time with changes in disease activity, chemotherapy, and supportive care. Without data extending beyond one year, it remains uncertain whether the favorable balance between efficacy and safety observed here persists in patients requiring multiyear anticoagulation. Longitudinal studies with extended observation windows are therefore needed to clarify the durability of these outcomes (6).

Finally, both trials primarily enrolled patients with solid tumors, good performance status, and preserved renal function, which limits extrapolation to those with hematologic malignancies, organ dysfunction, or frailty. The relatively homogeneous populations studied may underestimate the complexity and clinical variability of cancer-associated thrombosis encountered in routine care. Minor differences between studies in baseline cancer distributions and background therapies may also introduce residual heterogeneity that cannot be entirely mitigated by statistical pooling. Collectively, these factors highlight the importance of cautious interpretation when applying these findings to high-risk or underrepresented patient groups. Future research should aim to include more diverse cancer subtypes, particularly hematologic and metastatic cases, and incorporate stratified analyses by tumor biology, treatment exposure, and thrombosis phenotype to inform more personalized anticoagulation strategies in oncology.

### Future directions

Future research should focus on defining which subsets of patients derive the greatest benefit from dose de-escalation strategies. Large, multicenter, prospective studies with extended follow-up are needed to assess whether reduced-dose apixaban remains safe and effective beyond the 12-month horizon observed in EVE and API-CAT. Furthermore, studies should evaluate real-world outcomes in populations underrepresented in current trials, including those receiving intensive chemotherapy, patients with hematologic cancers, and those with significant renal impairment. Integration of biomarker-based risk stratification and predictive modeling may also help refine anticoagulation intensity over time, aligning therapy with evolving cancer status and treatment exposure.

## Conclusion

In patients with cancer-associated venous thromboembolism, reduced-dose apixaban (2.5 mg twice daily) appears to provide comparable protection against thromboembolic recurrence as the standard 5 mg regimen, with a lower incidence of clinically relevant non-major bleeding. These findings reinforce the safety and practicality of dose reduction after the initial 6-month treatment period for appropriately selected patients. However, the safety advantage may vary depending on cancer type, treatment intensity, and comorbidity profile. Extended follow-up and stratified trial designs are required to better define optimal de-escalation criteria and confirm long-term outcomes. Ultimately, the goal of future research should be to move toward precision-guided anticoagulation in oncology, balancing efficacy and safety to meet the individual needs of this fragile and heterogeneous population.

## Data Availability

The original contributions presented in the study are included in the article/**Supplementary Material**. Further inquiries can be directed to the corresponding author.
