# Supplementary material for: Reduced versus standard dose apixaban for secondary prevention of cancer-associated venous thromboembolism: A systematic review and meta-analysis
Source: Front Oncol. 2025 Dec 19;15:1690984. doi: 10.3389/fonc.2025.1690984 (PMC12757263; doi:10.3389/fonc.2025.1690984)
Supplement: Supplementary file 1 [file DataSheet1.pdf]

Supplemental Table of Contents

1. **Search Strategy** .... *Pages 2-5*
   1.1 PubMed Search
   .... *Page 2*
   1.2 EMBASE Search
   .... *Pages 2-3*
   1.3 CENTRAL Search
   .... *Page 4*

   1.4 ClinicalTrials.gov Search
   .... *Page 4*

   1.5 Web of Science Search
   .... *Pages 4-5*

2. **PRISMA Checklist**

   .... *Page 5-7*

3. **Risk of Bias Assessment (ROB 2.0)**
   .... *Page 7-8*

4. **GRADE Assessment Table**
   .... *Page 8-10*

1. **Search Strategy**

1.1 PubMed Search

Search Term:
((((((("apixaban" [Supplementary Concept]) OR (apixaban)) OR (eliquis)) OR (apixaban 2.5 mg)) OR (apixaban 2.5mg)) OR (apixaban 5mg)) OR (apixaban 5 mg)) OR (apixaban reduced dose)) AND ((((((((("Venous Thromboembolism"[Mesh]) OR (vte)) OR (dvt)) OR (thrombosis)) OR (venous thrombosis)) OR (venous thrombus)) OR (thrombus)) OR (deep vein thrombus)) OR (deep vein thrombosis)) AND ((("Neoplasms"[Mesh]) OR (cancer)) OR (cancer patients)))

- **Total Results:** 459 articles
- **Search Date:** November 5th, 2025

1.2 EMBASE Search

Search Term:
(('neoplasm'/exp OR 'acral tumor' OR 'acral tumour' OR 'neoplasia' OR 'neoplasm' OR 'neoplasms' OR 'neoplastic disease' OR 'neoplastic entity' OR 'neoplastic mass' OR 'tumor' OR 'tumoral entity' OR 'tumoral mass' OR 'tumorous entity' OR 'tumorous mass' OR 'tumors' OR 'tumour' OR 'tumoural entity' OR 'tumoural mass' OR 'tumourous entity' OR 'tumourous mass' OR 'tumours') OR ('malignant neoplasm'/exp OR 'cancer' OR 'cancers' OR 'malignant neoplasia' OR 'malignant neoplasm' OR 'malignant neoplastic disease' OR 'malignant tumor' OR 'malignant tumour' OR 'neoplasia, malignant' OR 'neoplasmic malignancy' OR 'neoplastic malignancy' OR 'oncologic malignancy' OR 'oncological malignancy' OR 'tumor, malignant' OR 'tumoral malignancy' OR 'tumorous malignancy' OR 'tumour, malignant') OR 'cancer patients') AND (('apixaban'/exp OR '1 (4 methoxyphenyl) 7 oxo 6 [4 (2 oxo 1 piperidinyl) phenyl] 1, 4, 5, 6, 7 pentahydropyrazolo [3, 4 c] pyridine 3 carboxamide' OR '1 (4 methoxyphenyl) 7 oxo 6 [4 (2 oxo 1 piperidinyl) phenyl] 4, 5, 6, 7 tetrahydro 1h pyrazolo [3, 4 c] pyridine 3 carboxamide' OR '1 (4 methoxyphenyl) 7 oxo 6 [4 (2 oxo 1 piperidinyl) phenyl] 4, 5, 6, 7 tetrahydropyrazolo [3, 4 c] pyridine 3 carboxamide' OR '1 (4 methoxyphenyl) 7 oxo 6 [4 (2 oxo 1 piperidyl) phenyl] 1, 4, 5, 6, 7 pentahydropyrazolo [3, 4 c] pyridine 3 carboxamide' OR '1 (4 methoxyphenyl) 7 oxo 6 [4 (2 oxo 1 piperidyl) phenyl] 4, 5, 6, 7 tetrahydro 1h pyrazolo [3, 4 c] pyridine 3 carboxamide' OR '1 (4 methoxyphenyl) 7 oxo 6 [4 (2 oxo 1 piperidyl) phenyl] 4, 5, 6, 7 tetrahydropyrazolo [3, 4 c] pyridine 3 carboxamide' OR '1 (4 methoxyphenyl) 7 oxo 6 [4 (2 oxopiperidin 1 yl) phenyl] 1, 4, 5, 6, 7 pentahydropyrazolo [3, 4 c] pyridine 3 carboxamide' OR '1 (4 methoxyphenyl) 7 oxo 6 [4 (2 oxopiperidin 1 yl) phenyl] 4, 5, 6, 7 tetrahydro 1h pyrazolo [3, 4 c] pyridine 3 carboxamide' OR '1 (4 methoxyphenyl) 7 oxo 6 [4 (2 oxopiperidin 1 yl) phenyl] 4, 5, 6, 7 tetrahydropyrazolo [3, 4 c] pyridine 3 carboxamide' OR '4, 5, 6, 7 tetrahydro 1 (4 methoxyphenyl) 7 oxo 6 [4 (2 oxo

1 piperidinyl) phenyl] 1 hydropyrazolo [3, 4 c] pyridine 3 carboxamide' OR '4, 5, 6, 7 tetrahydro 1 (4 methoxyphenyl) 7 oxo 6 [4 (2 oxo 1 piperidinyl) phenyl] 1h pyrazolo [3, 4 c] pyridine 3 carboxamide' OR '4, 5, 6, 7 tetrahydro 1 (4 methoxyphenyl) 7 oxo 6 [4 (2 oxo 1 piperidinyl) phenyl] pyrazolo [3, 4 c] pyridine 3 carboxamide' OR '4, 5, 6, 7 tetrahydro 1 (4 methoxyphenyl) 7 oxo 6 [4 (2 oxo 1 piperidyl) phenyl] 1 hydropyrazolo [3, 4 c] pyridine 3 carboxamide' OR '4, 5, 6, 7 tetrahydro 1 (4 methoxyphenyl) 7 oxo 6 [4 (2 oxo 1 piperidyl) phenyl] 1h pyrazolo [3, 4 c] pyridine 3 carboxamide' OR '4, 5, 6, 7 tetrahydro 1 (4 methoxyphenyl) 7 oxo 6 [4 (2 oxopiperidin 1 yl) phenyl] 1 hydropyrazolo [3, 4 c] pyridine 3 carboxamide' OR '4, 5, 6, 7 tetrahydro 1 (4 methoxyphenyl) 7 oxo 6 [4 (2 oxopiperidin 1 yl) phenyl] 1h pyrazolo [3, 4 c] pyridine 3 carboxamide' OR '4, 5, 6, 7 tetrahydro 1 (4 methoxyphenyl) 7 oxo 6 [4 (2 oxopiperidin 1 yl) phenyl] pyrazolo [3, 4 c] pyridine 3 carboxamide' OR 'aboxoma' OR 'apixaban' OR 'apixaben' OR 'bms 562247' OR 'bms 562247 01' OR 'bms 562247-01' OR 'bms562247' OR 'bms562247 01' OR 'bms562247-01' OR 'eliques' OR 'eliquis' OR 'lunast' OR 'pf 0465257' OR 'pf0465257' OR 'tah 3311' OR 'tah 3341' OR 'tah3311' OR 'tah3341') OR 'apixaban 2.5' OR 'apixaban reduced dose') AND (('venous thromboembolism'/exp OR 'thromboembolism, venous' OR 'vein thromboembolism' OR 'venous thromboembolism') OR ('deep vein thrombosis'/exp OR 'DVT (deep vein thrombosis)' OR 'acute DVT' OR 'acute deep venous thrombosis' OR 'deep thrombo-phlebitis' OR 'deep thrombophlebitis' OR 'deep vein blood clots' OR 'deep vein thrombophlebitis' OR 'deep vein thrombosis' OR 'deep vein thrombus' OR 'deep venous thrombophlebitis' OR 'deep venous thrombosis' OR 'deep venous thrombus' OR 'recurrent DVT' OR 'thrombosis, acute deep venous') OR ('lung embolism'/exp OR 'chronic lung embolism' OR 'embolism, lung' OR 'lung embolism' OR 'lung embolization' OR 'lung embolus' OR 'lung embolus recurrence' OR 'lung emboly' OR 'lung microembolism' OR 'lung microembolization' OR 'lung microembolus' OR 'lung thromboembolism' OR 'microembolus, lung' OR 'pulmonary embolism' OR 'pulmonary embolization' OR 'pulmonary embolus' OR 'pulmonary microembolism' OR 'pulmonary thromboembolic disease' OR 'pulmonary thromboembolism' OR 'thromboembolism, lung')) AND (('randomized controlled trial'/exp OR 'controlled trial, randomized' OR 'randomised controlled study' OR 'randomised controlled trial' OR 'randomized controlled study' OR 'randomized controlled trial' OR 'trial, randomized controlled') OR ('cohort analysis'/exp OR 'analysis, cohort' OR 'cohort analysis' OR 'cohort fertility' OR 'cohort life cycle' OR 'cohort studies' OR 'cohort study' OR 'fertility, cohort') OR ('observational study'/exp OR 'non experimental studies' OR 'non experimental study' OR 'nonexperimental studies' OR 'nonexperimental study' OR 'observation studies' OR 'observation study' OR 'observational studies' OR 'observational studies as topic' OR 'observational study' OR 'observational study as topic'))

- **Total Results:** 979 articles
- **Search Date:** November 5th, 2025

1.3 CENTRAL Search

Search Term:
(apixaban OR eliquis OR "apixaban 2.5 mg" OR "apixaban 5 mg" OR "reduced dose apixaban") AND ("venous thromboembolism" OR "venous thrombosis" OR "deep vein thrombosis" OR DVT OR VTE OR thrombosis OR thrombus OR "pulmonary embolism") AND (cancer OR neoplasm* OR malignan* OR tumor OR tumour OR oncolog*)

- **Total Results:** 198 articles
- **Search Date:** November 5th, 2025

1.4 ClinicalTrials.gov Search

Search Term:
apixaban OR eliquis OR "apixaban 2.5 mg" OR "apixaban 5 mg" OR "reduced dose apixaban" AND ("venous thromboembolism" OR VTE OR "deep vein thrombosis" OR DVT OR "pulmonary embolism" OR thrombosis) AND (cancer OR neoplasm OR malignancy OR tumor OR tumour OR oncology)

- **Total Results:** 501 articles
- **Search Date:** November 5th, 2025

1.5 Web of Science Search

Search Term:
ALL=(apixaban)

AND ALL=("venous thromboembolism" OR "deep vein thrombosis" OR DVT OR VTE OR thrombosis)

AND ALL=(cancer OR neoplasm OR malignancy OR tumor OR tumour OR oncology)

- **Total Results:** 675 articles
- **Search Date:** November 5th, 2025

Final Included Studies

- **Total Studies:** 2 RCTs

- **Studies Included:**
    - EVE 2024
    - API-CAT 2025

PRISMA Checklist

Section	Item	Checklist Item	Reported in Manuscript (Page/Section)
**TITLE**	1	Identify the report as a systematic review, meta-analysis, or both.	Title Page
**ABSTRACT**	2	Provide a structured abstract including background, objectives, data sources, study eligibility, participants, interventions, appraisal, synthesis methods, results, limitations, conclusions, and registration number.	Abstract
**INTRODUCTION**	3	Describe the rationale for the review in the context of existing knowledge.	Introduction
	4	Provide an explicit statement of the objectives or questions addressed.	End of Introduction
**METHODS**	5	Indicate whether a review protocol exists and, if so, provide registration information.	Methods: Study Design (PROSPERO ID: CRD420251026337)
	6	Specify all databases, registers, and other sources searched, and dates of coverage.	Methods: Search Strategy
	7	Present the full search strategies for all databases.	Supplementary Materials
	8	Specify the inclusion and exclusion criteria for the review.	Methods: Eligibility Criteria
	9	Specify the methods used to decide whether a study met the inclusion criteria.	Methods: Study Selection

	10	Describe all information sources and the process for data collection.	Methods: Data Extraction
	11	Specify all variables for which data were sought and any assumptions made.	Methods: Data Extraction
	12	Describe methods used for assessing risk of bias of individual studies.	Methods: Risk of Bias
	13	Specify the principal summary measures (e.g., RR, HR) and effect model used.	Methods: Statistical Analysis
	14	Describe methods of handling data and combining results of studies.	Methods: Statistical Analysis
	15	Describe methods for assessing heterogeneity.	Methods: Statistical Analysis
	16	Specify any methods used to assess risk of reporting biases.	Methods: Risk of Bias (Egger's test omitted per Cochrane guidance)
	17	Describe methods for assessing certainty in the body of evidence.	Methods: Risk of Bias and GRADE
**RESULTS**	18	Provide a flow diagram showing study selection.	Figure 1 (PRISMA Flow Diagram)
	19	Present characteristics of included studies.	Results: Characteristics of Included Studies (Table 1)
	20	Present risk of bias assessments.	Supplementary Materials, Table S1
	21	Present results of individual studies and syntheses.	Results and Table 3
	22	Describe heterogeneity measures and sensitivity analyses.	Results: Pooled Outcomes and Supplementary
**DISCUSSION**	23	Summarize main findings, including the strength of evidence and relevance to key groups.	Discussion

	24	Discuss limitations of the evidence and the review process.	Discussion: Limitations
	25	Provide general interpretation of the results and implications for future research.	Discussion: Future Directions
**OTHER INFORMATION**	26	Describe sources of funding and role of funders.	Funding / Acknowledgment (if applicable)
	27	Provide a data availability statement.	To be added before References
	28	Declare any conflicts of interest.	Declarations Section

ROB (Risk of Bias) 2.0

Table S1. Risk of Bias and Quality Assessment of Included Studies Using the Cochrane RoB 2.0 Tool.

Author (Year)	D1: Confounding	D2: Selection of Participants	D3: Classification of Interventions	D4: Deviations from Intended Interventions	D5: Missing Data	Overall Risk of Bias
**McBane et al. (2024)**[16]	Low	Low	Low	Low	Low	Low
**Mahé et al. (2025)**[17]	Low	Low	Low	Low	Moderate	Low

Figure S1. Traffic light plot for risk of bias assessment (RoB 2.0) across included randomized controlled trials.

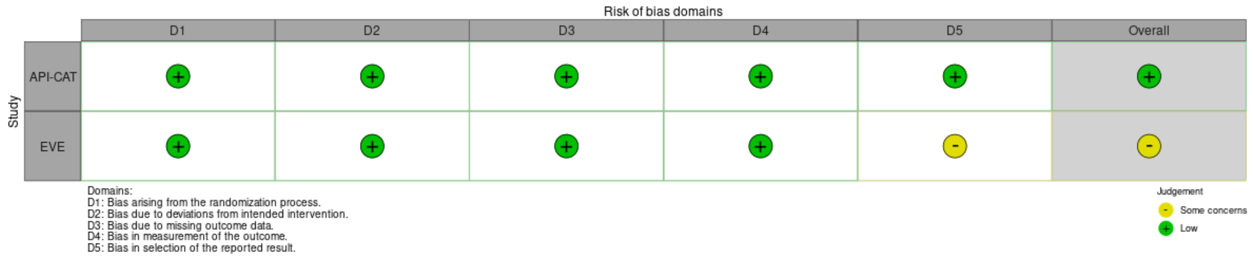

Figure S2. Summary plot of overall risk of bias (RoB 2.0) across included randomized controlled trials.

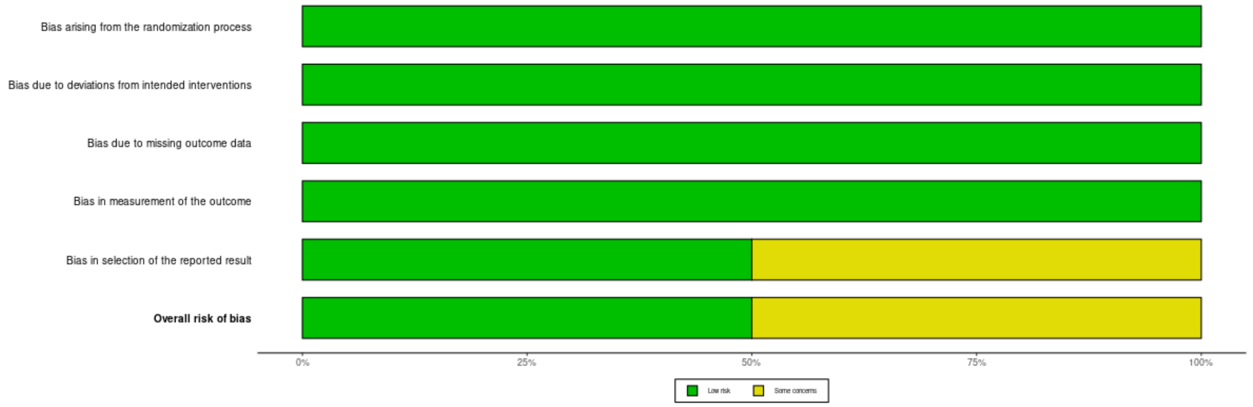

GRADE

Table S2. GRADE Evidence Profile of Included Outcomes

Outcome	Effect Estimate (95% CI)	I^2 (%)	Certainty of Evidence	Reasons for Rating
Composite Outcomes				
Recurrent VTE + Bleeding (RR)	0.79 (0.65 – 0.96)	0	High	No downgrades; adequate sample, CI excludes 1, consistent effect.
Major + Clinically Relevant Nonmajor Bleeding (HR)	0.63 (0.63 – 0.88)	0	High	No downgrades; narrow CI, consistent results across studies.

Individual Outcomes				
Recurrent VTE (HR)	0.83 (0.16 – 4.29)	0	Low	↓↓ for very serious imprecision; wide CI includes major benefit and harm.
Deep-Vein Thrombosis (RR)	0.95 (0.44 – 2.08)	0	Moderate	↓ for imprecision; CI crosses 1 with possible benefit or harm.
Pulmonary Embolism (RR)	0.89 (0.43 – 1.87)	0	Moderate	↓ for imprecision; low event rates and wide CI.
Major Bleeding (HR)	0.72 (0.05 – 11.13)	0	Low	↓↓ for very serious imprecision; sparse events and extremely wide CI.
Clinically Relevant Nonmajor Bleeding (HR)	0.77 (0.36 – 1.63)	0	Moderate	↓ for imprecision; CI includes benefit and harm.
All-Cause Mortality (HR)	0.96 (0.66 – 1.40)	0	High	No downgrades; objective endpoint, adequate precision, consistent results.

Footnotes for Table S2

a. Risk of bias: both trials were randomized, double blind, with independent adjudication and low attrition. No downgrades.

b. Inconsistency: I^2 equals 0 for all outcomes with overlapping confidence intervals and aligned point estimates. No downgrades.

c. Indirectness: study populations, interventions, and outcomes directly match the review question. No downgrades.

d. Imprecision: downgrades applied when confidence intervals crossed 1.0 and included effect sizes compatible with important benefit and harm or when event counts were sparse leading to very wide intervals despite a total sample size of about two thousand.

e. Publication bias: not assessed because only two studies were available which is below the recommended minimum for reliable small study bias testing.
